# Dual dimensions of sensitization: age-stratified and disease-associated sIgE profiles in pediatric eczema and urticaria

**DOI:** 10.3389/fnut.2026.1763720

**Published:** 2026-03-09

**Authors:** Liu Yang, Jiajia Ni, Zhiwei Zhu, Ci Li, Xiang Feng, Nan Chen, Panpan Fang, Junmei Yang, Kaijie Gao

**Affiliations:** 1 Zhengzhou Key Laboratory of Children's Infection and Immunity, Department of Clinical Laboratory, Children's Hospital Affiliated to Zhengzhou University, Zhengzhou, Henan, China; 2 Research and Development Center, Guangdong Meilikang Bio-Science Ltd, Foshan, Guangdong, China

**Keywords:** allergens, children, eczema, immunoglobulin E, urticaria

## Abstract

**Objective:**

To characterize sensitization patterns of allergen-specific immunoglobulin E (sIgE) in children with eczema and urticaria, compare differences by disease type and age group, and provide insights for prevention and treatment strategies.

**Methods:**

A single-center, retrospective cross-sectional study was performed using serum samples from 4,925 children diagnosed with eczema or urticaria at Henan Children’s Hospital between January 2022 and December 2023. Levels of sIgE to inhaled and food allergens were measured using fluorescence immunoassay. Group differences in sensitization rates were compared using chi-square tests, and Spearman correlation analysis was applied to assess relationships between sIgE levels and clinical parameters.

**Results:**

The overall positive rate of sIgE was 58.05%, with the positive rate for food allergens (52.93%) significantly higher than that for inhalant allergens (14.05%) (*p* < 0.001). The main allergen profiles exhibited age-dependent patterns: the sensitization rates for inhalant allergens (dust mite mix, mold mix, inhalant mix) increased with age, while the sensitization rates for food allergens (food mix, egg white, milk) were higher in the younger age group (under 3 years old). The unadjusted comparison showed differences in sensitization profiles: the positive rate of food allergens in children with eczema was significantly higher than that in children with urticaria (55.69% vs. 49.32%, *p* < 0.001), with an unadjusted odds ratio of 1.291. The sensitization rate of inhaled allergens in children with urticaria was significantly higher than that in children with eczema (25.90% vs. 7.57%, *p* < 0.001), with an unadjusted odds ratio of 0.234. Children with eczema and urticaria mainly exhibited low-level sIgE sensitization. High-grade (≥grade 4) positivity was uncommon and primarily associated with specific inhalant allergens. The correlation analysis between sIgE and clinical indicators showed that sIgE to food allergens (such as egg white, wheat, and food mixture) in children with eczema was significantly positively correlated with eosinophil counts, while sIgE to inhalant allergens (such as *Dermatophagoides pteronyssinus*, *Dermatophagoides farinae*, mixed dust mite, and mixed grasses) in children with urticaria was significantly positively correlated with hemoglobin concentration.

**Conclusion:**

This study identified distinct age-dependent sIgE sensitization profiles, while disease-associated differences were attenuated after accounting for age and sex. These findings highlight age as a primary dimension for sensitization risk stratification and underscore the clinical value of integrating sIgE monitoring with other biomarkers, such as eosinophils and hemoglobin.

## Introduction

The global incidence of allergic skin diseases in children has risen steadily (1, 2), making eczema and urticaria substantial public health concerns due to their considerable impact on quality of life (3, 4). Eczema is characterized by dry skin, erythema, pruritus, and inflammation, with pathogenesis linked to skin barrier dysfunction, immune dysregulation, and environmental triggers (5). Urticaria typically presents with wheals, pruritus, and angioedema, primarily mediated by IgE-associated type I hypersensitivity reactions (6). Children exhibit heightened allergen sensitivity compared to adults, likely due to immune immaturity (7–9). Eczema and urticaria, despite their distinct pathophysiologies, are among the most prevalent allergic skin conditions in children. In clinical practice, both frequently necessitate evaluation for allergen sensitization. However, large-scale studies directly comparing their sensitization profiles have often failed to adequately control for key demographic confounders, particularly age and sex (10, 11). Therefore, a systematic assessment of the independent association between disease type and allergen-specific immunoglobulin E (sIgE) sensitization patterns-after controlling for these confounders-is essential. Such an analysis is crucial to clarify the true determinants of sensitization and to inform evidence-based clinical management.

This large-scale, cross-sectional study aimed to evaluate differences in sIgE sensitization profiles between children with eczema and urticaria in Central China, with adjustment for age and sex. The analysis encompassed sensitization to specific allergen types, age-dependent patterns, and correlations with key hematological parameters. The findings provide valuable insights to guide targeted prevention and personalized treatment strategies in this region.

## Methods

### Study design and setting

This was a single-center, retrospective study conducted at Henan Children’s Hospital. We analyzed the clinical and laboratory data of pediatric patients diagnosed with eczema or urticaria between January 1, 2022, and December 31, 2023.

### Ethical considerations

The study protocol was reviewed and approved by the Ethics Committee of Henan Children’s Hospital (No: 2024-k-027). Given that this study is a retrospective study, the data collected have removed the patient-related privacy information and informed consent is waived.

### Study population and eligibility criteria

Children aged 18 years or younger who visited the hospital during the study period were considered. Inclusion criteria was: (1) a clinical diagnosis of eczema or urticaria made by a pediatric dermatologist in accordance with the Chinese guidelines for diagnosis and treatment of eczema (12) and urticaria (13), respectively; and (2) availability of serum-specific immunoglobulin E (sIgE) test results. Exclusion criteria was: (1) diagnosis of primary or secondary immunodeficiency; (2) use of systemic corticosteroids or immunosuppressants within 4 weeks prior to blood sampling; (3) incomplete medical records regarding key variables (diagnosis, age, sIgE results); and (4) for patients with multiple visits, only the first record was included.

### Data collection and variable definitions

Two trained researchers independently extracted data from electronic medical records using a pre-designed, standardized case report form. Extracted data included demographics (age, sex), diagnosis, sIgE profiles, and complete blood count parameters. Any discrepancy was resolved by a third senior clinician. For this study, sensitization was strictly defined as an sIgE level ≥0.35 kUA/L. The term clinical allergy was reserved for conditions involving both sensitization and documented reproducible symptoms, which was not systematically assessed in this retrospective design.

### Measurement of allergen-specific IgE

Serum levels of sIgE were quantified using the Phadia 250 automated fluorescent immunoassay system (Thermo Fisher Scientific, USA). The tested allergen panel was a standardized combination covering the most common food (e.g., egg white, milk) and inhalant (e.g., house dust mite, mold mix) allergens in Chinese children, selected based on the epidemiological data of common allergens in Chinese children and the combination determined by the clinical practice guidelines of this region. In accordance with international standards, an sIgE concentration ≥0.35 kUA/L was defined as positive (14).

### Statistical analysis

Statistical analyses were performed using SPSS 22.0 (IBM Corp., USA). Categorical variables were presented as *n* (%) and compared using the Chi-square test. Continuous variables were described as median (IQR) and compared using non-parametric tests. Spearman’s correlation was used for correlation analysis. To control for potential confounders, multivariable binary logistic regression models were employed. The outcome variables were sensitization status to food or inhalant allergens. The primary independent variable was disease type (eczema vs. urticaria), adjusted for age group and sex. Results were expressed as adjusted odds ratios (aOR) with 95% confidence intervals (CI). A two-sided *p*-value < 0.05 was considered significant.

## Results

### Overall sensitization profile

This cohort consisted of 3,157 children diagnosed with eczema and 1,768 with urticaria, comprising 2,933 boys (aged 1 month to 16 years) and 1,992 girls (aged 29 days to 17 years). Patients were stratified by age as follows: <1 year (*n* = 2,794; 56.73%), 1–3 years (*n* = 1,019; 20.69%), 3–6 years (*n* = 601; 12.20%), and >6 years (*n* = 511; 10.38%) (Table 1). Specific IgE (sIgE) testing was performed for all participants. The overall sIgE sensitization rate was 58.50% (95% CI: 56.85–60.15). Sensitization to food allergens was observed in 52.93% (95% CI: 51.54–54.33) of children, while sensitization to inhalant allergens was found in 14.05% (95% CI: 13.08–15.02). The most commonly detected food allergen sIgE were against food mix (46.15%; 95% CI: 44.68–47.63), egg white (37.36%; 95% CI: 36.00–38.73), and milk (30.14%; 95% CI: 28.85–31.43). Among inhalant allergens, the highest sensitization rates were for dust mite mix (14.24%; 95% CI: 10.25–18.23), mold mix (12.78%; 95% CI: 11.61–13.96), and inhalation mix (12.65%; 95% CI: 11.43–13.87).

**Table 1:** Characteristics of participants.

Item	Number	Percentage (%)
Age group		
<1 year	2,794	56.73
1–3 years	1,019	20.69
3–6 years	601	12.20
>6 years	511	10.38
Sex		
Male	2,933	59.55
Female	1,992	40.45
Clinical diagnosis		
Eczema	3,157	64.10
Urticaria	1,768	35.90

### Relationship between allergen positive rate and gender

Among the 4,925 children, the allergen positivity rate was 59.26% (1,738/2,933) in boys and 57.38% (1,143/1,992) in girls. The food allergen positivity rate was 54.52% (1,599/2,933) in boys and 51.76% (1,031/1,992) in girls. The inhalant allergen positivity rate was 14.49% (425/2,933) in boys and 13.65% (272/1,992) in girls. No statistically significant differences were observed in food allergen, inhalant allergen, or overall allergen positivity rates between genders (Table 2).

**Table 2:** Comparison of the positive rates of allergens in different genders [*n*(%)].

Gender	Numbers	Positive for any single allergen	Positive for food allergens	Positive for inhalation allergens
Male	2,933	1,738 (59.26)	1,599 (54.52)	425 (14.49)
Female	1,992	1,143 (57.38)	1,031 (51.76)	272 (13.65)
*χ* ^2^		1.722	3.633	0.682
*P*		0.189	0.057	0.409

### Relationship between allergen positivity rates and age

**Figure 1:**
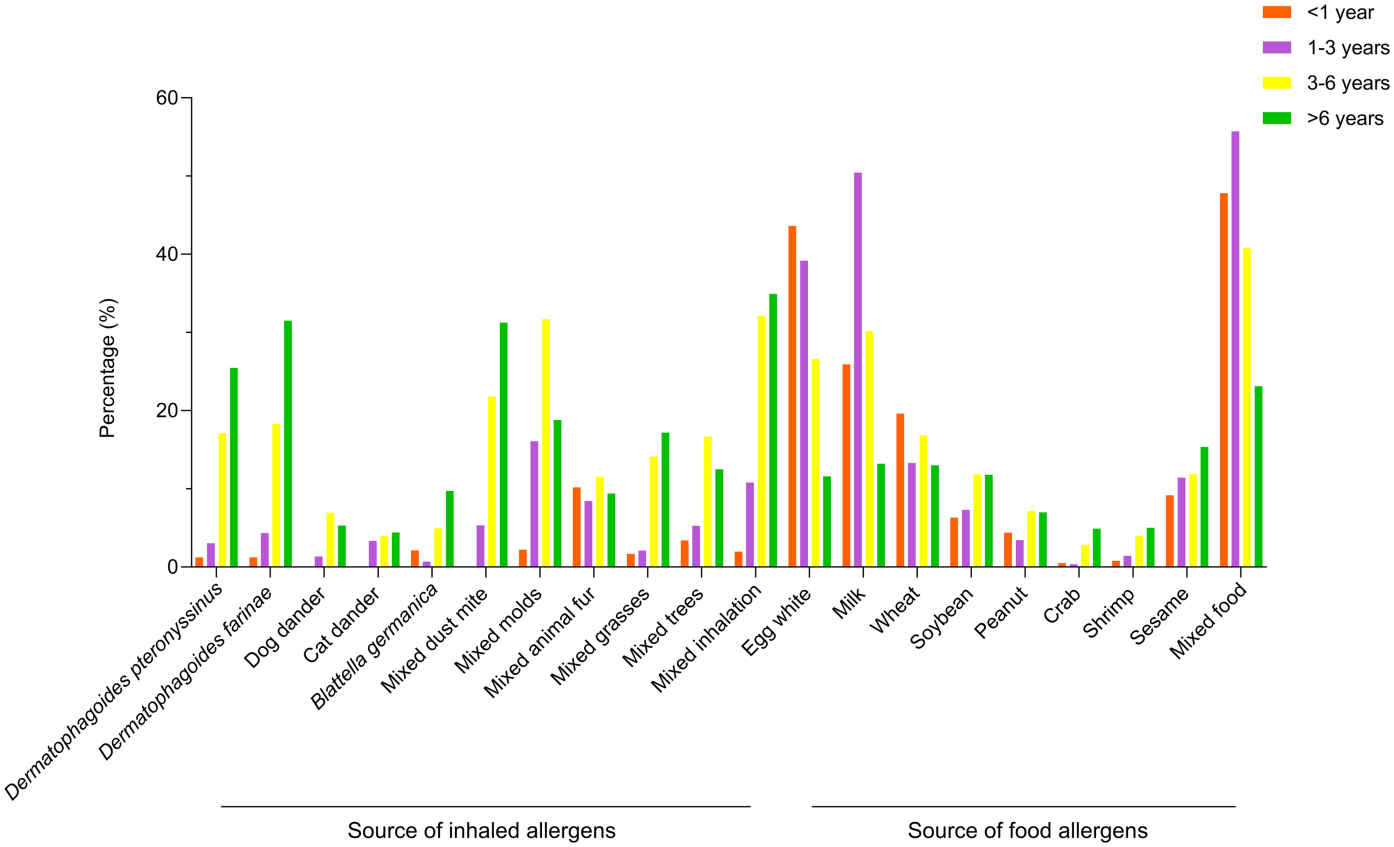
Sensitization rates to inhaled and food allergens across age groups. The bars show the percentage of children sensitized to major allergens in different age groups: <1 year, 1–3 years, 3–6 years, and >6 years. Each segment represents a specific allergen.

Among the different age groups, statistically significant differences were observed for all allergens except cat dander, mixed animal fur, and sesame (Table 3 and Figure 1).

**Table 3:** Comparison of allergen positive rates in different ages [*n*(%)].

Sources of allergens	<1 year (*n* = 2,794)	1–3 years (*n* = 1,019)	3–6 years (*n* = 601)	>6 years (*n* = 511)	*χ* ^2^	*p*
Positive for food allergens	1,559 (55.80%)	621 (60.94%)	287 (47.75%)	163 (31.90%)	132.389	<0.001
Positive for inhalation allergens	58 (2.08%)	187 (18.35%)	253 (42.10%)	199 (38.94%)	994.952	<0.001
*Dermatophagoides pteronyssinus*	4 (1.22%)	7 (3.03%)	26 (17.11%)	42 (25.46%)	100.85	<0.001
*Dermatophagoides farinae*	4 (1.22%)	10 (4.33%)	26 (18.31%)	52 (31.52%)	124.871	<0.001
Dog dander	0 (0.00%)	2 (1.34%)	7 (6.93%)	6 (5.31%)	10.88	0.012
Cat dander	0 (0.00%)	5 (3.36%)	4 (3.96%)	5 (4.43%)	4.153	0.245
*Blattella germanica*	2 (2.13%)	1 (0.69%)	5 (4.95%)	11 (9.74%)	14.134	0.003
Mixed dust mite	0 (0.00%)	5 (5.32%)	17 (21.79%)	20 (31.25%)	34.736	<0.001
Mixed molds	31 (2.22%)	128 (16.10%)	163 (31.71%)	75 (18.80%)	325.989	<0.001
Mixed animal fur	6 (10.17%)	8 (8.42%)	9 (11.54%)	6 (9.38%)	0.493	0.92
Mixed grasses	1 (1.69%)	2 (2.11%)	11 (14.10%)	11 (17.19%)	17.969	<0.001
Mixed trees	2 (3.39%)	5 (5.26%)	13 (16.67%)	8 (12.50%)	9.913	0.019
Mixed inhalation	27 (1.97%)	76 (10.81%)	140 (32.11%)	117 (34.93%)	443.71	<0.001
Egg white	1,209 (43.63%)	389 (39.17%)	155 (26.59%)	58 (11.60%)	218.635	<0.001
Milk	718 (25.91%)	501 (50.45%)	176 (30.19%)	66 (13.20%)	286.261	<0.001
Wheat	544 (19.63%)	132 (13.29%)	98 (16.81%)	65 (13.00%)	28.222	<0.001
Soybean	175 (6.32%)	72 (7.29%)	69 (11.84%)	59 (11.80%)	33.221	<0.001
Peanut	122 (4.40%)	34 (3.42%)	42 (7.20%)	35 (7.00%)	17.716	0.001
Crab	7 (0.50%)	1 (0.35%)	4 (2.80%)	8 (4.91%)	34.381	<0.001
Shrimp	22 (0.80%)	14 (1.42%)	23 (3.97%)	25 (5.02%)	62.481	<0.001
Sesame	128 (9.18%)	32 (11.43%)	17 (11.89%)	25 (15.34%)	7.178	0.066
Mixed food	1,209 (47.81%)	497 (55.72%)	217 (40.79%)	99 (23.13%)	133.051	<0.001

### Comparison of allergen sensitization profiles in children with eczema and urticaria

**Figure 2:**
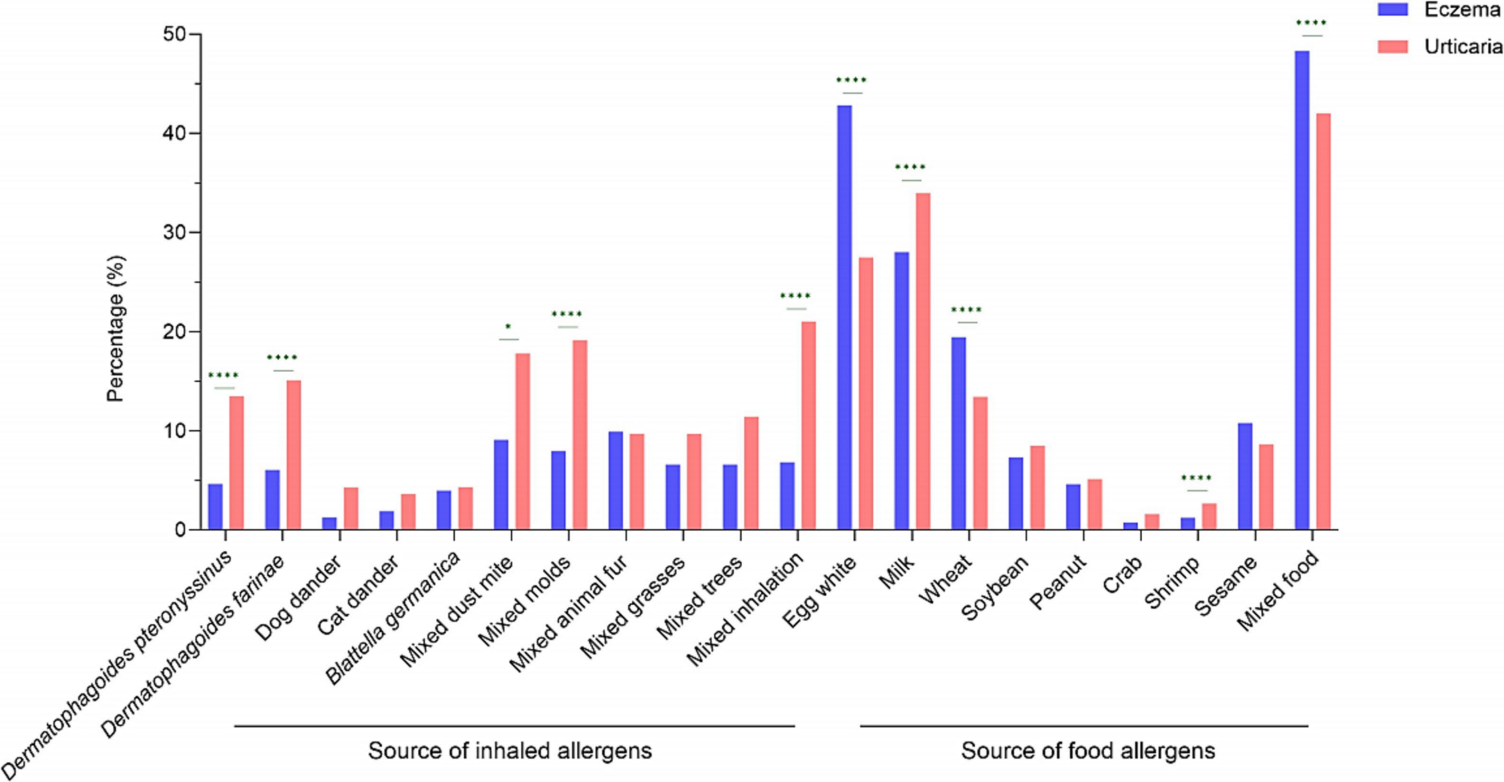
Comparison of sensitization rates between eczema and urticaria children. Prevalence of sensitization to food and inhaled allergens is compared. Group comparisons were made using the Chi-square test. ^*^p < 0.05; ^****^p < 0.0001.

Among 4,925 pediatric cases, 3,157 were diagnosed with eczema and 1,768 with urticaria. The overall sIgE positivity rate was similar between groups: 58.35% (1,842/3,157) in eczema patients and 58.77% (1,039/1,768) in urticaria patients. However, marked differences emerged in specific allergen responses. The inhaled allergen sIgE positivity rate was significantly higher in urticaria patients (25.90%, 458/1,768) than in eczema patients (7.57%, 239/3,157; *χ*^2^ = 313.569, *p* < 0.001). Conversely, food allergen sIgE positivity was more frequent in eczema patients (55.69%, 1,758/3,157) than in urticaria patients (49.32%, 872/1,768; *χ*^2^ = 18.448, *p* < 0.001), as shown in Table 4. The predominant allergens also differed between conditions. In eczema patients, the top five sensitizations were mixed food (48.32%), egg white (42.83%), milk (28.02%), wheat (19.46%), and sesame (10.80%). Among urticaria patients, the most common were mixed food (42.01%), milk (33.97%), egg white (27.49%), mixed inhalation (21.03%), and mixed molds (19.16%). Significant intergroup differences in sIgE positivity were observed for multiple specific allergens, including *Dermatophagoides pteronyssinus*, *Dermatophagoides farinae*, mixed dust mite, mixed molds, mixed inhalation, egg white, milk, wheat, shrimp, and mixed food (*p* < 0.05; Figure 2). Both groups predominantly exhibited low-grade sIgE sensitization. High-grade reactions (≥grade 4) were infrequent and primarily directed against *Dermatophagoides pteronyssinus, Dermatophagoides farinae,* mixed dust mite, mixed molds, mixed grasses, and mixed inhalation. There were statistically significant differences in the positive rate of grade 4 or above allergens between children with eczema and urticaria, including *Dermatophagoides farinae*, mixed molds, mixed inhalation, egg white, wheat, and mixed food (Supplementary Table S1).

**Table 4:** Comparison of positive rates of allergens in children with eczema and urticaria [*n*(%)].

Clinical diagnosis	Numbers	Positive for food allergens	Positive for inhalation allergens
Eczema	3,157	1,758 (55.69%)	239 (7.57%)
Urticaria	1,768	872 (49.32%)	458 (25.90%)
*χ* ^2^		18.448	313.569
*P*		<0.001	<0.001

### Association between allergen sIgE sensitization and clinical profiles in pediatric eczema and urticaria

**Figure 3:**
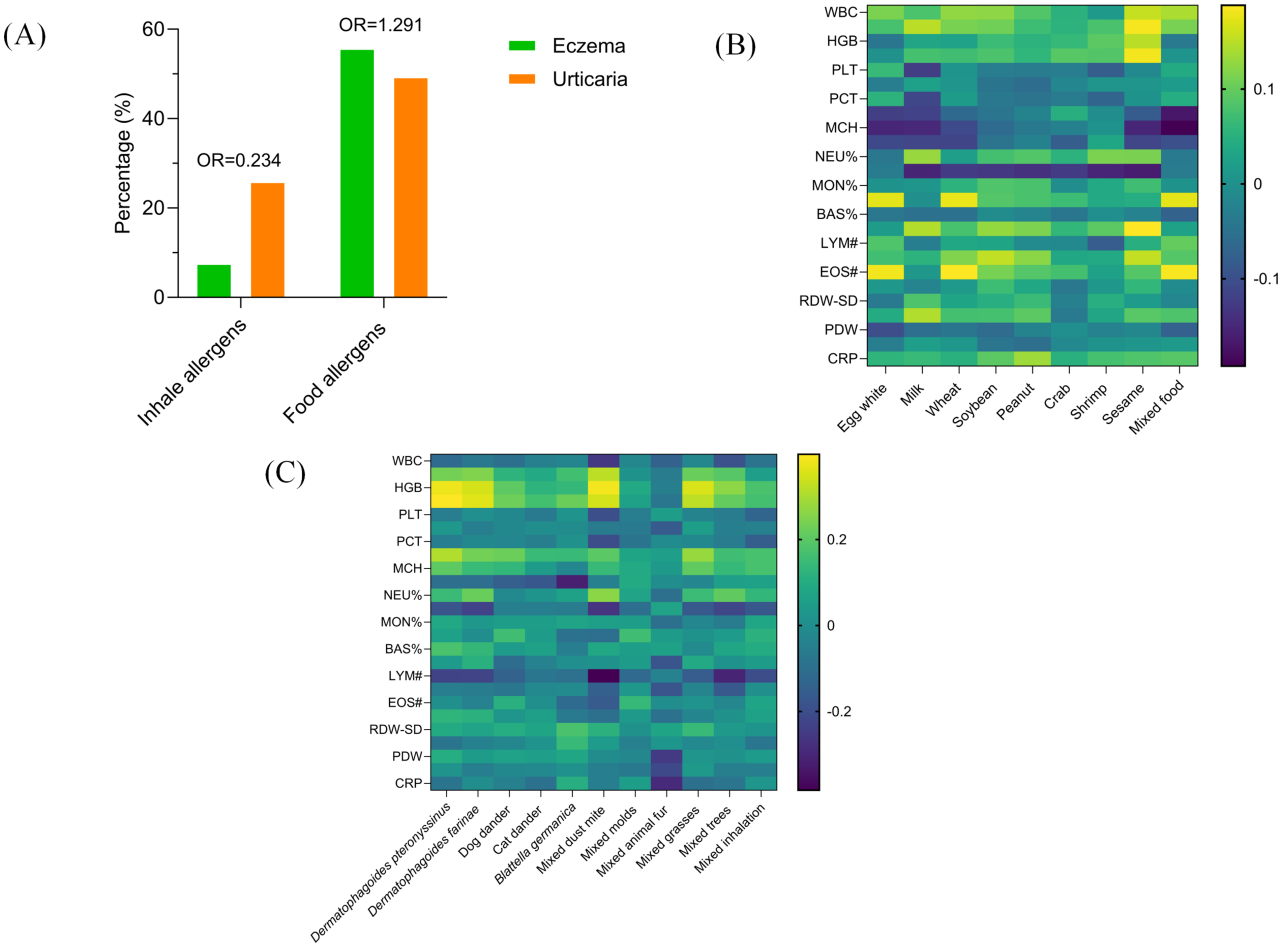
Sensitization patterns and clinical correlations in pediatric eczema and urticaria. **(A)** Bar chart comparing the prevalence of sensitization to food and inhalant allergens between children with eczema and urticaria. Odds ratios (OR) with 95% confidence intervals are presented to quantify the association between disease type and sensitization risk. **(B)** Heatmap of Spearman’s rank correlation coefficients between levels of specific food sIgE and selected hematological parameters in the eczema group. **(C)** Heatmap of Spearman’s rank correlation coefficients between levels of specific inhalant sIgE and selected hematological parameters in the urticaria group.

Initial unadjusted comparisons revealed differences in sensitization patterns between the two diseases. Eczema patients showed a higher prevalence of food allergen sensitization than urticaria patients (55.69% vs. 49.32%; unadjusted OR = 1.29), whereas urticaria patients had a higher prevalence of inhalant allergen sensitization (25.90% vs. 7.57%; unadjusted OR = 0.23) (Figure 3A). To assess the independent association of disease type while controlling for the strong confounding effects of age and sex, multivariable logistic regression analyses were performed (Table 5). For food allergen sensitization, age was the dominant independent risk factor. Compared to children under 1 year, those aged 1–3, 3–6, and >6 years had significantly higher adjusted risks (aORs = 2.69, 3.35, and 1.96, respectively; all *p* < 0.001). After this adjustment, disease type was no longer a significant predictor (aOR = 0.99, 95% CI: 0.86–1.15; *p* = 0.916). For inhaled allergens sensitization, risk declined sharply with age beyond infancy (e.g., aOR for 1–3 years = 0.03, 95% CI: 0.02–0.04; *p* < 0.001), and male sex was protective (aOR = 0.71, 95% CI: 0.59–0.85; *p* < 0.001). After adjusting for these factors, disease type also showed no independent association (aOR = 1.10, 95% CI: 0.89–1.36; *p* = 0.386). Analysis of correlations between food allergens sIgE levels and hematological parameters in pediatric eczema patients found several significant correlations. Specific allergens were positively correlated with eosinophil counts: egg white (*R* = 0.181, *p* < 0.001), wheat (*R* = 0.187, *p* < 0.001), and food mix (*R* = 0.185, *p* < 0.001). Peanut sIgE levels showed a positive correlation with monocyte count (*R* = 0.153, *p* < 0.001). Conversely, sIgE levels for multiple allergens were inversely correlated with lymphocyte percentage: milk (*R* = −0.155, *p* < 0.001), soybean (*R* = −0.140, *p* < 0.001), crab (*R* = −0.126, *p* = 0.021), shrimp (*R* = −0.154, *p* < 0.001), and sesame (*R* = −0.164, *p* = 0.003) (Figure 3B). In urticaria patients, inhaled allergens exhibited distinct hematological associations. Multiple allergens showed positive correlations with hemoglobin concentration: *Dermatophagoides pteronyssinus* (*R* = 0.378, *p* < 0.001), *Dermatophagoides farinae* (*R* = 0.349, *p* < 0.001), mixed dust mite (*R* = 0.384, *p* = 0.002), and mixed grasses (*R* = 0.353, *p* = 0.004). Positive associations with eosinophil percentage were observed for mixed molds (*R* = 0.157, *p* < 0.001) and mixed inhalation (*R* = 0.116, *p* = 0.004). Negative correlations with lymphocyte count were identified for *Dermatophagoides pteronyssinus* (*R* = −0.229, *p* = 0.002), *Dermatophagoides farinae* (*R* = −0.227, *p* = 0.003), mixed dust mite (*R* = −0.383, *p* = 0.002), and mixed trees (*R* = −0.307, *p* = 0.013) (Figure 3C).

**Table 5:** Factors associated with sensitization to food and inhaled allergens: results of multivariable logistic regression analyses.

Variable	Category	Food allergens sensitization		Inhalation allergens sensitization	
		aOR (95% CI)	*P*	aOR (95% CI)	*P*
Age group	<1 year (Ref)	1.00	–	1.00	–
	1–3 years	2.69 (2.15–3.38)	<0.001	0.03 (0.02–0.04)	<0.001
	3–6 years	3.35 (2.66–4.22)	<0.001	0.34 (0.27–0.44)	<0.001
	>6 years	1.96 (1.53–2.51)	<0.001	1.14 (0.90–1.46)	0.276
Disease type	Eczema (Ref)	1.00	–	1.00	–
	Urticaria	0.99 (0.86–1.15)	0.916	1.10 (0.89–1.36)	0.386
Sex	Female (Ref)	1.00	–	1.00	–
	Male	0.90 (0.80–1.01)	0.083	0.71 (0.59–0.85)	<0.001

aOR, adjusted odds ratio; CI, confidence interval; Ref, reference category.

## Discussion

Eczema and urticaria represent common allergic dermatoses in pediatric populations, originating from a complex interplay between genetic susceptibility, environmental factors, and immune dysregulation. The substantial diversity and often subtle presentation of allergens frequently complicate clinical management, leading to diagnostic uncertainties and suboptimal avoidance measures (15). Consequently, precise characterization of regional sensitization pattern, particularly dynamic distributions across age groups and disease subtypes, is essential for advancing diagnostic and therapeutic approaches (14, 16). The most significant insight from this study is the observation regarding the differences in allergen sensitization profiles between eczema and urticaria, achieved by controlling for confounders through multivariable analysis. While unadjusted comparisons suggested disease-specific patterns, our analysis suggests that these observed differences were primarily attributable to disparities in the age and sex composition of the patient groups, rather than to the distinct pathophysiologies of the diseases themselves. This finding highlights the importance of adjusting for fundamental demographic confounders when comparing allergic phenotypes across different disease cohorts. Taken together, these results suggest a hypothesis that that developmental stage (age) may be a primary determinant of sensitization risk, rather than disease type alone.

This retrospective investigation examined clinical records of 4,925 children diagnosed with eczema or urticaria at Henan Children’s Hospital from January 2022 to December 2024. Through comprehensive allergen profiling using the ImmunoCAP system, we delineated sensitization characteristics and contributing factors in pediatric allergic dermatitis. The analysis demonstrated an overall allergen-specific IgE positivity rate of 58.50%. Food allergens exhibited markedly higher sensitization prevalence (53.40%) compared to inhaled allergens (14.15%), consistent with established literature (17, 18). This disparity may reflect the predominance of children under 3 years in our cohort, whose developing gastrointestinal systems demonstrate impaired protein digestion and enhanced macromolecular permeability that potentially promotes sensitization (19). Among food allergens, the highest reactivity was observed to mixed food (46.15%), followed by egg white (37.36%) and milk (30.14%). Primary inhaled allergens included dust mite mix (14.24%), mold mix (12.78%), and inhaled mix (12.65%). These findings underscore substantial geographical variation in sensitization patterns. While egg white and dust mites emerge as fundamental triggers in pediatric sensitization, regional climate conditions and dietary habits appear to significantly modulate sensitization profiles (20, 21). For example, Shanghai pediatric populations demonstrate *Dermatophagoides farinae/pteronyssinus* sensitization rates reaching 80.94%, indicating strong climate dependency associated with elevated ambient humidity (22). Additionally, Singapore-Malaysia Chinese cohort data establish that high-fat dietary patterns characterized by frequent consumption of fried foods and processed meats substantially increase risk of sensitization (23). Collectively, these observations emphasize how diverse environmental exposures shape distinct allergen reactivity profiles.

Age suggests a profound influence on allergen sensitization profiles, with distinct patterns emerging across pediatric developmental stages. Our analysis reveals a transition from food allergen predominance in children under 6 years to inhalant allergen dominance in older children. Food allergen sIgE sensitization peaked at 60.94% in children aged 1–3 years, likely reflecting expanded dietary diversity and increased antigen exposure. The subsequent development of food tolerance coincides with gastrointestinal maturation (24), while rising inhalant sensitization after age three correlates with increased outdoor activities, consistent with established epidemiological patterns (25). Age-specific sensitization profiles revealed egg white as the predominant allergen in infants (43.63%, <1 year), milk in toddlers (50.45%, 1–3 years), and mixed inhalation in both preschool (32.11%, 3–6 years) and school-aged children (34.93%, >6 years). Statistical analysis demonstrated significant age-dependent variations for all allergens except cat dander, mixed animal fur, and sesame. This progression aligns with the classical “allergic march” concept: immature intestinal barrier function and frequent dietary antigen exposure during early life promote food-specific sIgE responses, while subsequent respiratory exposure and immunological maturation shift sensitization toward inhalant allergens (26, 27). Emerging evidence suggests early introduction of potentially allergenic foods may redirect this trajectory (28). Notably, peanut introduction at 4–6 months reduces sensitization risk by 86% in high-risk populations (29, 30). These findings suggest that the high food sensitization prevalence observed in our young cohort might be addressed through optimized complementary feeding strategies for primary prevention, though this hypothesis requires confirmation in prospective studies.

Analysis of gender differences revealed modestly elevated sensitization rates in male children across all allergen categories (overall: 59.26% vs. 57.38%; food allergens: 54.52% vs. 51.76%; inhaled allergens: 14.49% vs. 13.65%), aligning with previous reports by Beutner et al. (31). However, these intergender differences did not achieve statistical significance (all *p*-values > 0.05). The gender disparity in food allergen sensitization approached but did not reach significance (*p* = 0.057), with minimal effect size indicating the observed variation likely represents sampling variation rather than meaningful biological differences. Consequently, our findings do not support gender as a significant determinant of allergen sensitization in this pediatric cohort. Future studies should incorporate larger sample sizes and adjust for potential confounders including age and environmental exposures to validate these observations.

Distinct sensitization profiles were observed between the two conditions, with eczema patients showing significantly higher food allergen sensitization (55.69% vs. 49.32%) and urticaria patients exhibiting greater inhalant allergen reactivity (25.90% vs. 7.57%). However, an important limitation must be acknowledged when interpreting these comparisons: due to the retrospective nature of our data, we were unable to subclassify urticaria into acute versus chronic, spontaneous versus inducible, or IgE-mediated versus non-IgE-mediated forms. This is a critical consideration, as a substantial proportion of pediatric urticaria, particularly chronic cases, may involve non-IgE-mediated mechanisms. Therefore, the sIgE sensitization patterns observed in our urticaria cohort likely reflect a heterogeneous group and should be interpreted with this caveat in mind. With this understanding, children with eczema demonstrated eosinophil-associated food sensitization patterns, particularly to egg white, wheat, and mixed food, which correlated positively with eosinophil counts. This aligns with established Th2-mediated pathways, where eosinophils serve as key effector cells in food-triggered eczema. The findings further support the role of these allergens in promoting eosinophilic inflammation, potentially via cytokines including IL-5 and IL-13 that stimulate bone marrow release and cutaneous infiltration of eosinophils, thereby perpetuating chronic inflammation in eczema (26, 32). In contrast, urticaria children showed predominant sensitization to inhalant allergens, which may be IgE-mediated immediate hypersensitivity mechanisms in a subset of patients. Inhalant antigens such as dust mites and molds directly bind mast cell-surface IgE, initiating histamine release and wheal formation (33). Negative correlations between sensitization to specific inhalant allergens (*Dermatophagoides pteronyssinus*, *Dermatophagoides farinae*, mixed molds, and mixed trees) and lymphocyte counts may indicate possible lymphocyte apoptosis or tissue migration induced by inhalant allergens, leading to peripheral lymphocytopenia, though this remains speculative. Analysis of high-grade (≥class 4) sensitization revealed significantly higher rates to *Dermatophagoides farinae*, mixed molds, and mixed inhalation among urticaria, potentially reflecting direct mast cell activation via respiratory mucosal exposure. Notable differences in egg white and wheat sensitization further hint at cross-reactive inflammatory activation by specific food antigens (34, 35). The eczema group exhibited no single predominant allergen but demonstrated lower mixed food sensitization rates, which may indicate a pattern of polyantigen-driven low-grade chronic inflammation. These findings may support dietary antigen avoidance in eczema management (36, 37), while suggesting the importance of environmental control and anti-IgE therapies for some forms of urticaria, though definitive clinical recommendations would require further studies (38, 39).

This study provides a characterization of the patterns of sIgE sensitization in a large pediatric cohort from Central China, offering hypothesis-generating evidence for regional clinical practice. Several limitations must be acknowledged. First, the single-center, retrospective design may introduce selection bias and limit the generalizability of the findings. Second, and most critically, the retrospective nature of data collection imposed inherent constraints: we were unable to distinguish between sensitization and clinical allergy, lacked standardized disease activity measures such as SCORAD scores, and—importantly—could not subclassify urticaria into acute versus chronic, spontaneous versus inducible, or IgE-mediated versus non-IgE-mediated forms. This inability to address urticaria heterogeneity is particularly significant, as a substantial proportion of pediatric urticaria involves non-IgE-mediated mechanisms. Consequently, the sIgE findings presented here should not be generalized to all urticaria subtypes, and the clinical interpretability of disease-specific comparisons is inherently weakened by this limitation. Third, from a statistical perspective, the multiple comparisons performed increase the risk of false-positive findings; therefore, while the core comparative findings are robust, isolated significant correlations should be interpreted with caution. Future prospective studies designed to systematically collect comprehensive clinical data, including validated activity scores and detailed urticaria subphenotyping, are needed to develop more precise diagnostic and preventive strategies.

## Conclusion

This study delineates distinct sensitization profiles in a large pediatric cohort with eczema or urticaria. Age emerged as the dominant and independent determinant of sensitization risk, with food allergens peaking in early childhood and inhalant sensitization showing a unique, declining prevalence after infancy. While unadjusted comparisons suggested disease-specific patterns, multivariable analysis revealed that these differences were largely attributable to underlying variations in the age and sex distribution between the groups, rather than to the disease entity itself. Therefore, clinical assessment for allergic sensitization in these children should prioritize age-stratified approaches. The combined assessment of sIgE with other clinical parameters may facilitate precise prevention and treatment strategies, ultimately improving quality of life in affected children.

## Data Availability

The original contributions presented in the study are included in the article/Supplementary material, further inquiries can be directed to the corresponding authors.
