# Supplementary material for: Dual dimensions of sensitization: age-stratified and disease-associated sIgE profiles in pediatric eczema and urticaria
Source: Front Nutr. 2026 Mar 9;13:1763720. doi: 10.3389/fnut.2026.1763720 (PMC13006257; doi:10.3389/fnut.2026.1763720)
Supplement: Supplementary file 1 [file Table_1.DOCX]

Supplementary materials

Dual Dimensions of Sensitization: Age-Stratified and Disease-Specific sIgE Profiles in Pediatric Eczema and Urticaria

Liu Yang^1^, Jiajia Ni^2^, Zhiwei Zhu^1^, Ci Li^1^, Xiang Feng^1^, Nan Chen^1^, Panpan Fang^1^, Junmei Yang^1*^, Kaijie Gao^1*^

^1^Department of Clinical Laboratory, Children's Hospital Affiliated to Zhengzhou University, Zhengzhou Key Laboratory of Children's Infection and Immunity, Zhengzhou, Henan, People's Republic of China; ^2^Research and Development Center, Guangdong Meilikang Bio-Science Ltd, Foshan, Guangdong People's Republic of China

*Correspondence: Junmei Yang (yangjunmei7683@163.com) and Kaijie Gao (welcomtogkj@126.com)

Table S1 Comparison of positive rates of allergens above Grade 4 in children with eczema and urticaria.

| Sources of allergens | Eczema(n=3157) | Urticaria(n=1768) | χ^2^ | *P* |
| --- | --- | --- | --- | --- |
| *Dermatophagoides pteronyssinus* | 4（20.00%） | 10（13.50%） | 2.502 | 0.114 |
| *Dermatophagoides farinae* | 6（23.08%） | 18（15.10%） | 5.946 | 0.015* |
| Dog dander | 0（0.00%） | 1（4.30%） | 0.528 | 0.468 |
| Cat dander | 0（0.00%） | 1（3.64%） | 0.528 | 0.468 |
| *Blattella germanica* | 0（0.00%） | 0（4.32%） | / | / |
| Mixed dust mite | 2（18.18%） | 7（17.82%） | 1.355 | 0.244 |
| Mixed molds | 16（11.35%） | 40（19.16%） | 18.785 | 0.001** |
| Mixed animal fur | 1（8.33%） | 0（9.71%） | 1.451 | 0.228 |
| Mixed grasses | 1（12.50%） | 5（9.71%） | 1.485 | 0.223 |
| Mixed trees | 0（0.00%） | 3（11.43%） | 2.096 | 0.148 |
| Mixed inhalation | 15（13.04%） | 48（21.03%） | 33.121 | <0.001*** |
| Egg white | 106（7.93%） | 8（27.49%） | 41.723 | <0.001*** |
| Milk | 23（2.63%） | 12（33.97%） | 0.028 | 0.867 |
| Wheat | 44（7.25%） | 4（13.43%） | 15.77 | <0.001*** |
| Soybean | 4（1.75%） | 7（8.52%） | 3.754 | 0.053 |
| Peanut | 3（2.08%） | 1（5.15%） | 0.198 | 0.656 |
| Crab | 0（0.00%） | 0（1.62%） | / | / |
| Shrimp | 1（2.63%） | 2（2.68%） | 1.247 | 0.264 |
| Sesame | 9（5.84%） | 1（8.66%） | 1.612 | 0.204 |
| Mixed food | 60（4.31%） | 12（42.01%） | 10.085 | 0.001** |

**Notes:** Chi-square test: **p* <0.05, ***p* <0.01, ****p* <0.001.
